# European spiny lobster recovery from overfishing enhanced through active restocking in Fully Protected Areas

**DOI:** 10.1038/s41598-019-49553-8

**Published:** 2019-09-10

**Authors:** Alessandro Cau, Andrea Bellodi, Rita Cannas, Maurizio Fois, Paolo Guidetti, Davide Moccia, Cristina Porcu, Antonio Pusceddu, Maria C. Follesa

**Affiliations:** 1 Dipartimento di Scienze della Vita e dell’Ambiente, Università di Cagliari, Via Tommaso Fiorelli 1, 09126 Cagliari, Italy; 2 Université Côte d’Azur, CNRS, FRE 3729 ECOMERS, Parc Valrose 28, Avenue Valrose, 06108 Nice, France; 3 Consorzio Interuniversitario per le Scienze del Mare, CoNISMa, Piazzale Flaminio 9, 00196 Rome, Italy

**Keywords:** Conservation biology, Conservation biology, Marine biology, Marine biology

## Abstract

Fully protected areas (FPAs) help preserving biodiversity and reversing the global decline of fishery resources. Stocks of the European spiny lobster *Palinurus elephas* (Fabr. 1787), among the most precious *gourmet* seafood worldwide, are currently facing a dramatic decline. Previous attempts of recovery based on fishery restrictions or active post-larval restocking in marine reserves provided unsuccessful outcomes. Here we present results of a 5-year restocking program carried through a Collaborative Fishery Research (CFR) project, in three *ad-hoc* established FPAs replenished using below-legal size wild juveniles. Results showed that Catch per Unit Effort (CPUE) in terms of both density and biomass burst (by ca. 300–700%) just 2 years since FPAs establishment, regardless of location. We also report tangible spillover effects (ca. 30–50% increase in density and biomass CPUE outside the FPAs) by the end of the program. Data from a 15-years lasting monitoring of a pilot FPA established in 1998, where the restocking protocol was conducted and protection kept in force once restocking ceased, demonstrated the persistence in time of restocking’ benefits. We foster that creation of FPAs assisted with local restocking under oriented CFR programs can represent an option for the recovery of European spiny lobster stocks from overfishing.

## Introduction

The decline of fishery resources is a matter of fact worldwide^1,2^. Currently, 77% of global fish stocks are overfished and this percentage is predicted to possibly increase up to 88% by 2050, causing also dramatic declines in ecosystem functions and services^1,3,4^. What is still under debate is whether this trend can be somehow reversed^5^.

Small Scale Fishery (SSF) refers to a wide array of fishery practices and is often associated with other terms such as: artisanal, local, coastal, traditional, small, non-industrial or low-tech fishery^6^. For instance, in Europe, SSF allude to fisheries carried out with below 12 m length vessels^6^. SSF represents ca. 20% of global catches and generates the most tangible food supply and/or economic revenue for coastal communities^7^; thus, overexploitation of coastal resources due to SSF is a critical issue worldwide. As a consequence, similarly to any other fishery^8^, SSF is experiencing a global crisis that weights on societal costs through subsidies for the support of SSF underperformance^9^.

Crustaceans belonging to the family *Palinuridae* are among the most highly priced seafood in the world and, in some regions (*e*.*g*., Caribbean countries), do represent the backbone of export economy, employing thousands of people and producing millions of dollars of annual income^10^. Fishery of the European spiny lobster *Palinurus elephas* (Fabr. 1787) dates back to Romans age (starting from 15^th^ century BC)^11^ and its popularity as *gourmet* food in modern times started in the 19^th^ century, continuously increasing till present days. To date, living specimens of *P*. *elephas* can be sold at European market prices ranging between 40 and 120 € per Kg^12^. As a general trend, *P*. *elephas* stocks faced a drastic decline in the second half of the 20^th^ century, particularly in the Mediterranean Sea, leading this species to be ranked as Vulnerable in the IUCN red list^13^.

Conservation measures do need places where nature is left wild. In the most protective end of the conservation tools spectrum, there are Fully Protected Areas (FPAs), which are equivalent to No-Take marine Reserves (NTRs), where any consumptive activity on marine stocks is prohibited^14^. FPAs are expected to protect many quantitative and compositional components of biodiversity and, at the same time, to potentially help overexploited stocks to recover^15–17^. However, assuming that FPAs are invariably effective in fisheries management is an unjustified overgeneralization^18^. Moreover, FPAs effectiveness is often blurred by factors such as: (i) the lack of a proper spatial and temporal replication in the sampling design^16,19,20^; (ii) improper features of the reserve itself (*e*.*g*., extension, location and enforcement^21–23,24^; (iii) the reliability of data coming from fishermen, especially if these data are provided by SSF operators^25^. These biases can result in concluding that FPAs do not target the whole set of conservation objectives which ultimately foster stakeholders’ scepticism about their reliability and efficacy^19,26^. In this regard, a recently proposed tool to overcome stakeholders’ scepticism is represented by Collaborative Fishery Research (CFR) programs, by which fishermen actively assist scientists in all phases of research. CFR programs allow indeed: (i) sharing methods, approaches and goals^19^; (ii) facilitating the social acceptance of fishery rules by increasing the scientific awareness of fishermen; (iii) reducing enforcement costs by improving the social compliance to the protection/restriction policies^27^.

European Union regulations such as the Common Fisheries Policy (European Commission [EC] no. 1380/2013) and Marine Strategy Framework Directive (2008/56/EC) now require the implementation of an ecosystem approach to marine management. In this context, a question has been raised as to whether a management strategy based on combining *ad hoc* established FPAs and active restocking using wild juveniles of *P*. *elephas* could enhance the recovery of this valuable resource (sometimes requiring decade^28^) and, nonetheless, induce tangible benefits for neighbouring fishing grounds. To provide insights on this topic, we investigated the variations in terms of Catch-Per-Unit-Effort in density (nCPUE) and biomass (CPUE) of European spiny lobster stocks in three FPAs around the island of Sardinia (Mediterranean Sea), restocked for five consecutive years with below legal-size specimens caught outside the FPAs through a dedicated CFR program. The effect provided by active restocking was discriminated by the single effect of protection through a comparison with a FPA where restocking was not performed, while the persistence in time of observed benefits coming from this experiment was investigated through a long-term investigation in a pilot FPA established in 1998^29^, where the introduction of wild juveniles was stopped after 5 years and fishing ban was kept in force.

## Results

**Figure 1:**
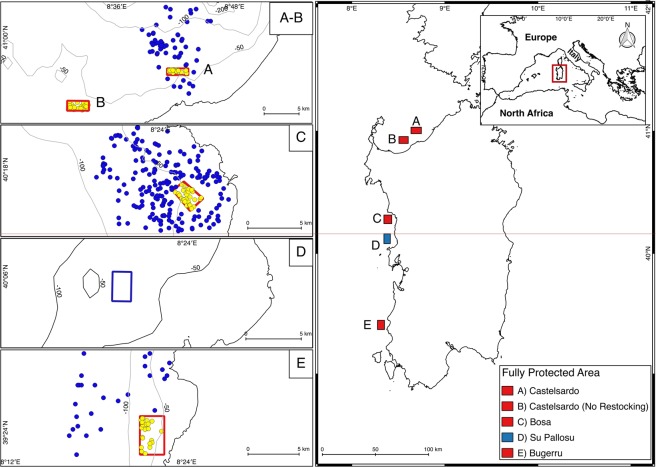
Map of the study area. Yellow dots represent replicates (*i*.*e*., set of trammel nets) performed inside FPAs, where fishing activities are prohibited. Blue dots represent set performed outside FPAs, in neighboring fishing grounds comprised within a 5 nautical miles radius from FPAs’ center, where below-legal size lobsters were released. The map was generated using QGIS software, version 2.18. 64 bit; https://www.qgis.org/.

During the five years of investigation, a total of 540 replicates (*i*.*e*., sets of trammel nets) have been conducted in the 3 FPAs (Fig. 1, Table 1), and 8,213 below legal-size specimens of *P*. *elephas* were tagged and released at the centre of each of the 3 FPAs since 2011. All details regarding the number of replicates and number of spiny lobsters introduced each year, per each FPA, are reported in Table 1. Details on the legal framework in which this program was carried out are provided in a dedicated section of methods.

**Table 1:** Area, depth range and geographical coordinates of the three FPAs object of the study, plus the pilot area of Su Pallosu and the Castelsardo FPA where restocking was not performed.

FPA	Area (km^2^)	Depth Range	replicates (n)		released lobsters (n)	Latitude (N)	Longitude (E)
Castelsardo	5.52	50–80 m	2010	15	—	40° 57′ 32″	8° 41′ 07″
			2011	27	870	40° 57′ 01″	8° 43′ 10″
			2012	12	693	40° 57′ 32″	8° 41′ 07″
			2013	15	302	40° 57′ 01″	8° 43′ 10″
			2014	10	36		
			2015	16	148		
Bosa	3.91	40–70 m	2010	4	—	40° 17′ 24″	8° 25′ 32″
			2011	17	145	40° 16′ 33″	8° 26′ 48″
			2012	90	864	40° 15′ 56″	8° 25′ 56″
			2013	88	725	40°16′ 58″	8° 24′ 48″
			2014	97	1309		
			2015	35	329		
Buggerru	7.8	40–70 m	2010	6	—	39° 24′ 50″	8° 20′ 49″
			2011	29	757	39° 24′ 52″	8° 22′ 29″
			2012	41	783	39° 22′ 50″	8° 22′ 29″
			2013	14	276	39° 22′ 52″	8° 20′ 49″
			2014	16	458		
			2015	33	518		
Castelsardo (no restocking)	5.50	50–80 m	2010	2	—	40° 54′ 19″	8° 30′ 55″
			2011	4	—	40° 55′ 70″	8° 30′ 56″
			2012	5	—	40° 55′ 60″	8° 33′ 90″
			2013	3	—	40° 54′ 21″	8° 33′ 90″
			2014	4	—		
			2015	8	—		
Su Pallosu (Pilot Area)	4.0	50–80 m	2007	36	—	40° 06′ 20″	8° 19′ 20″
			2008	186	—	40° 06′ 20″	8° 20′ 30″
			2009	57	—	40° 04′ 90″	8° 19′ 20″
			2010	47	—	40° 04′ 90″	8° 20′ 30″
			2011	110	—		
			2012	101	—		
			2013	32	—		

Reported are also the number of surveys conducted inside and outside each FPA in the five sampling years. Surveys were conducted during fishing season lasting from April 1^st^ to August 30^th^. With respect to the FPA of Su Pallosu, we report data from 2006 to 2013, while details regarding restocking performed in the first 5 years ca be found in Follesa *et al*.^29^.

**Figure 2:**
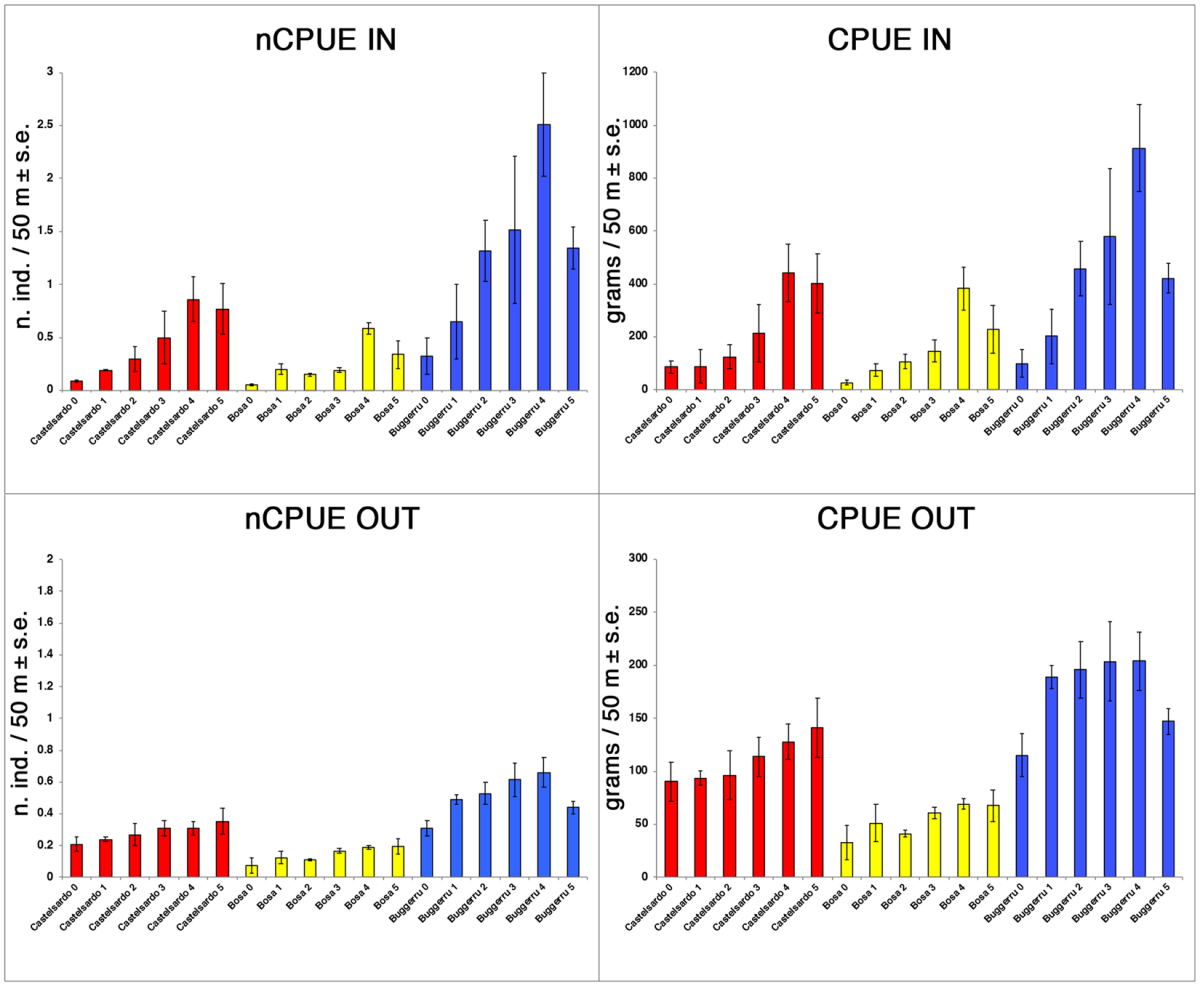
Plot showing annual CPUE (gr/50 m ± s.e.) and nCPUE trends (n. ind/50 m ± s.e.) inside (upper graph) and outside (lower graph) FPAs borders before the beginning (time zero) and during restocking (time 1, 2, 3, 4, 5).

Both CPUE and nCPUE varied between inner and outer portions of each FPA and with time (Tables 2, 3 and 4). The CPUE and nCPUE annual average increased significantly (up to eight folds) either inside or outside the FPAs until the fourth year, then slightly decreased in some cases during the fifth year (Fig. 2; Table 3).

**Table 2:** Output from the PERMANOVA analysis (main test). Significant Monte Carlo procedure p-values [P(MC)] are reported in bold.

CPUE					nCPUE				
Source	df	MS	Pseudo-F	P(MC)	Source	df	MS	Pseudo-F	P(MC)
**Castelsardo**									
Time	5	94.08	5.95	**0.001**	Time	5	94.08	5.95	**0.001**
FPA	1	163.36	10.32	**0.001**	FPA	1	163.36	10.32	**0.001**
Time × FPA	5	40.55	2.56	**0.032**	Time × FPA	5	40.55	2.56	**0.032**
Res	83	15.82			Res	83	15.82		
Total	94				Total	94			
**Bosa**									
FPA	1	0.19	34.619	**0.001**	FPA	1	0.196	8.265	**0.006**
Time	5	89125	16.195	**0.001**	Time	5	0.242	10.219	**0.001**
Time × FPA	5	62633	11.563	**0.001**	Time × FPA	5	0.132	5.568	**0.001**
Res	319	5503.2			Res	83			
Total	330				Total	94			
**Buggerru**									
FPA	1	747.56	39.63	**0.001**	FPA	1	9.46	52.86	**0.001**
Time	5	152.67	8.09	**0.001**	Time	5	1.84	10.27	**0.001**
Time × FPA	5	91.41	4.84	**0.001**	Time × FPA	5	1.05	5.88	**0.001**
Res	127	18.86			Res	127	0.179		
Total	138				Total	138

**Table 3:** Output from the pairwise comparison testing for differences in average CPUE among years in the inside (IN) and outside (OUT) portion of FPAs.

	CASTELSARDO IN							CASTELSARDO OUT					
	nCPUE							nCPUE					
CPUE	Time Zero	Time + 1	Time + 2	Time + 3	Time + 4	Time + 5	CPUE	Time Zero	Time + 1	Time + 2	Time + 3	Time + 4	Time + 5
Time Zero		0.594	0.345	0.295	0.069	0.235	Time Zero		0.465	0.415	0.132	0.186	0.081
Time + 1	0.973		0.5	0.277	**0.024**	0.145	Time + 1	0.837		0.439	0.092	**0.107**	0.032
Time + 2	0.636	0.6		0.388	**0.041**	0.176	Time + 2	0.838	0.883		0.689	**0.732**	0.444
Time + 3	0.432	0.269	0.404		0.31	0.522	Time + 3	0.399	0.271	0.575		0.99	0.588
Time + 4	0.097	**0.025**	**0.019**	0.217		0.819	Time + 4	0.236	**0.058**	**0.293**	0.634		0.603
Time + 5	0.214	0.084	0.091	0.349	0.835		Time + 5	0.15	0.034	0.026	0.406	0.697	
	**BOSA IN**							**BOSA OUT**					
	**nCPUE**							**nCPUE**					
**CPUE**	Time Zero	Time + 1	Time + 2	Time + 3	Time + 4	Time + 5	**CPUE**	Time Zero	Time + 1	Time + 2	Time + 3	Time + 4	Time + 5
Time Zero		0.106	0.06	0.1	0.067	0.289	Time Zero		0.669	0.508	0.367	0.202	0.518
Time + 1	0.208		0.325	0.928	**0.179**	0.615	Time + 1	0.813		0.607	0.336	**0.094**	0.331
Time + 2	0.177	0.62		0.362	**0.011**	0.187	Time + 2	0.791	0.754		0.004	**0.001**	0.007
Time + 3	0.206	0.462	0.56		0.034	0.345	Time + 3	0.454	0.285	0.008		0.318	0.457
Time + 4	0.018	**0.041**	**0.003**	0.027		0.156	Time + 4	0.257	**0.048**	**0.001**	0.123		0.837
Time + 5	0.13	0.314	0.187	0.444	0.114		Time + 5	0.484	0.31	0.014	0.67	0.569	
	**BUGGERRU IN**							**BUGGERRU OUT**					
	**nCPUE**							**nCPUE**					
**CPUE**	Time Zero	Time + 1	Time + 2	Time + 3	Time + 4	Time + 5	**CPUE**	Time Zero	Time + 1	Time + 2	Time + 3	Time + 4	Time + 5
Time Zero		0.522	0.094	0.257	0.038	0.047	Time Zero		0.023	0.32	0.11	0.035	0.075
Time + 1	0.458		0.295	0.414	**0.073**	0.166	Time + 1	0.005		0.653	0.123	**0.023**	0.002
Time + 2	0.113	0.284		0.787	**0.048**	0.95	Time + 2	0.455	0.514		0.531	**0.346**	0.047
Time + 3	0.14	0.265	0.63		0.271	0.754	Time + 3	0.25	0.957	0.73		0.727	0.008
Time + 4	0.009	**0.012**	**0.053**	0.222		0.021	Time + 4	0.049	**0.617**	**0.504**	0.775		**0.002**
Time + 5	0.015	0.087	0.935	0.465	0.007		Time + 5	0.068	0.001	0.025	0.009	0.001	

Significant Monte Carlo p-values (pMC < 0.05) differences are reported in bold.

**Table 4:** Output from the pairwise comparison testing for differences in annual average biomass (CPUE) and density (nCPUE) values between inside and outside of the three FPAs, across years.

CPUE			nCPUE		
	t	p(MC)		t	p(MC)
**CASTELSARDO**					
Time Zero	0.052	0.964	Time Zero	1.049	0.339
Time + 1	0.186	0.844	Time + 1	0.754	0.474
Time + 2	0.610	0.584	Time + 2	0.208	0.843
Time + 3	1.648	0.121	Time + 3	1.324	0.22
Time + 4	3.514	**0.011**	Time + 4	3.251	**0.006**
Time + 5	2.25	**0.045**	Time + 5	1.651	0.122
**BOSA**					
Time Zero	0.26	0.831	Time Zero	0.49	0.661
Time + 1	0.47	0.642	Time + 1	0.64	0.502
Time + 2	4.9	**0.001**	Time + 2	1.26	0.2
Time + 3	3.74	**0.001**	Time + 3	0.41	0.69
Time + 4	10.83	**0.001**	Time + 4	6.77	**0.001**
Time + 5	2.77	**0.009**	Time + 5	1.33	0.194
**BUGGERRU**					
Time Zero	0.466	0.659	Time Zero	0.116	0.917
Time + 1	0.699	0.945	Time + 1	1.263	0.224
Time + 2	2.91	**0.005**	Time + 2	3.831	**0.001**
Time + 3	2.6	**0.024**	Time + 3	2.366	**0.042**
Time + 4	7.31	**0.001**	Time + 4	5.401	**0.001**
Time + 5	7.92	**0.001**	Time + 5	7.464	**0.001**

Values of nCPUE and CPUE at the end of the study were 3–7.5 folds and 0.4–2 folds higher than those at the beginning, in FPAs and neighboring waters, respectively. Rates of both CPUE and nCPUE annual enhancement varied among the three FPAs, ranging between 10 and 160% and 3 and 67%, inside and outside the FPAs, respectively (Fig. 2).

**Figure 3:**
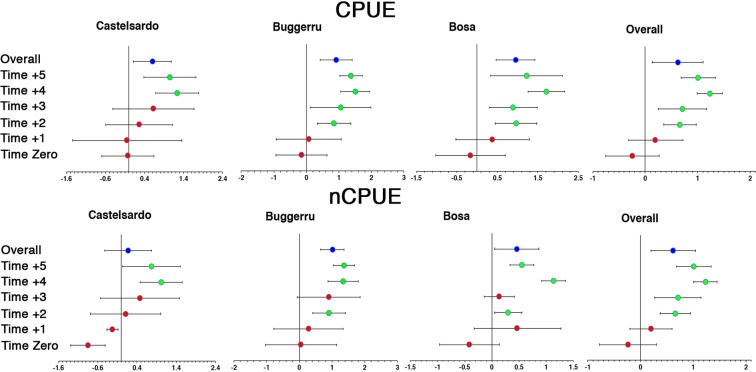
Forest Plot showing cumulative impacts of FPAs (ln–response ratio; mean ± 95% confidence intervals). Red circles: non-significant ratios; green circles: significant ratios; blue circles: overall ratio, across time. Cumulative effects are significant if confidence intervals do not overlap zero.

The active restocking with below legal-size specimens in the FPAs exerted a significantly positive effect on CPUE, regardless of lobsters’ size, just after 2 years in Buggerru (LnR = 0.85 ± 0.51; 95% CI) and Bosa (LnR = 0.96 ± 0.52; 95% CI) and after 4 years in Castelsardo (LnR = 1.24 ± 0.55; 95% CI) (Fig. 3).

A significantly positive effect was also observed for nCPUE after 2 years in Buggerru (LnR = 0.91 ± 0.5; 95% CI) and Bosa (LnR = 0.30 ± 0.25; 95% CI) while it was significant after 4 years in Castelsardo (LnR = 0.91 ± 0.5; 95% CI) (Fig. 3).

Pooling data from all locations together, the effect was significantly positive starting at year 2 both in terms of either CPUE or nCPUE (Fig. 3). The highest positive effects for CPUE were observed at year 4 in Bosa (LnR = 1.71 ± 0.45; 95% CI), Castelsardo (LnR = 1.24 ± 0.53; 95% CI; Fig. 3) and Buggerru (LnR = 1.50 ± 0.44; 95% CI). In terms of nCPUE the highest positive effects were observed at year 4 in Castelsardo (LnR = 1.02 ± 0.54; 95% CI), Bosa (LnR = 1.14 ± 0.22; 95% CI; Fig. 3) and at year 5 in Buggerru (LnR = 1.37 ± 0.67; 95% CI).

Biomass (CPUE) of legal size individuals (>90 mm of carapace length, CL) increased significantly in the neighboring areas of just one (Bosa) of three FPAs (PERMANOVA, p < 0.001), whereas density (nCPUE) of legal size individuals did not vary with time out of the FPAs.

**Figure 4:**
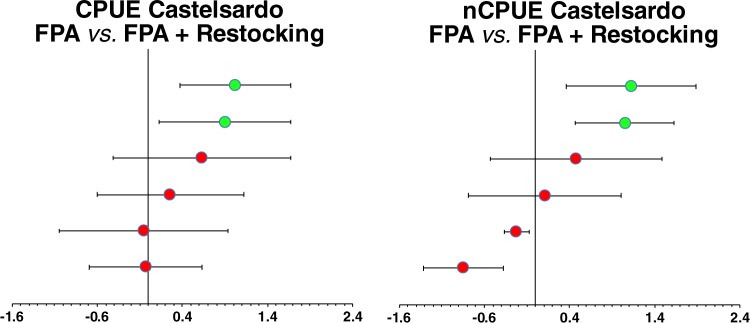
Forest Plot showing cumulative impacts of restocking in the two FPAs of Castelsardo (ln–response ratio; mean ± 95% confidence intervals). Red circles: non-significant ratios; green circles: significant ratios. Cumulative effects are significant if confidence intervals do not overlap zero.

To discriminate the positive effect exerted by active restocking from the one exerted by lobsters’ protection within FPA borders, we compared both density and biomass of the inner part of Castelsardo FPA with a reference FPA where restocking was not performed (see map in Fig. 1). The forest plot produced shows the positive effect of the cumulative effect of restocking and protection, being significantly higher than the single effect provided by protection after 4 years, both for nCPUE (LnR = 1.05 ± 0.58; 95% CI) and CPUE (LnR = 0.9 ± 0.77; 95% CI (Fig. 4).

**Figure 5:**
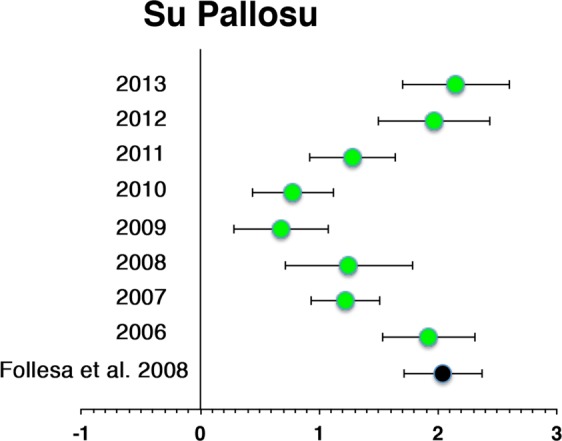
Forest Plot showing cumulative impacts in Su Pallosu FPA (ln–response ratio; mean ± 95% confidence intervals). Red circles: overall ratio reported in the pilot study started in 1998 and concluded in 2005; green circles: significant ratios from year 2006 to 2013.

The long-term analysis performed in the Su Pallosu FPA showed a positive and significant effect persisting also after restocking ceased (Fig. 5). In detail, the forest plot reports data from 2006 to 2013, while data regarding the restocking period from 1998 to 2003 and the following 2 years (2004 and 2005) have been aggregated into a single point. Original data from that study are reported in Follesa *et al*., 2008^29^. Despite the effect was positive and significant during the entire monitoring period, it decreased in magnitude from before 2006 to 2009 (Fig. 5). From 2009 onward, the effect increased again reaching a value (LnR = 2.15 ± 0.45; 95% CI; Fig. 5) comparable to that observed at the beginning of the monitoring period, when restocking was already ceased (in 2003).

Throughout the study period, the size class distribution range widened both inside and outside all FPAs. Inside Bosa FPA lobsters’ mean CL ranged between a maximum of 94.5 mm ± 22 mm at year 2 to a minimum of 88.4 ± 23 mm at year 4 and (Supp. Fig. 1) while outside the Bosa FPA it ranged between 84.86 mm ± 12.62 mm at year 0 and 72.9 ± 9.9 mm in year 5 (Supp. Fig. 1). Also, it was observed an increase in specimens that could be landed: from 2.06% in year 1 to 5.02% in year 5, and also the portion of individuals smaller than 60 mm CL, which were absent in the first sampling years (years 0 and 1), constantly increased from ca. 0.1% at year 2 to 7.4% at year 5 (Supp. Fig. 1).

Inside Buggerru FPA, the mean size of individuals remained quite constant across years, varying between 74.2 ± 13.6 mm at year 2 and 70 ± 11.9 mm during year 5. The same trend was observed outside, with mean CL varying between 76.9 ± 4.2 mm at year 1 and 72.4 ± 4.5 mm at year 4 (Supp. Fig. 2). Outside Buggerru FPA, size class composition was similar across years, with size classes ranging between 60 and 95 mm changing in frequency across years (Supp. Fig. 2). The portion of smaller specimens (CL 60–65 mm) that were not collected in the first years of sampling, constantly arose up to ca. 4%, cumulatively, at year 5 (Supp. Fig. 2).

Inside Castelsardo FPA, mean CL values ranged between 71.7 ± 22 mm at year 2 and 81.9 ± 11 mm at year 4 (Supp. Fig. 3) while outside FPA the minimum value was recorded at the beginning of the experiment (74.9 ± 4.5 mm) and the maximum was recorded during the last sampling year (80.6 ± 5 mm). The portion of large individuals increased from 7.3% during year 1 to ca. 16% and 12% at year 4 and 5, respectively (Supp. Fig. 3).

## Discussion

Ensuring the delivery of ecosystem goods and services in the face of rapid environmental changes and increasing human pressure on fishing stocks is, by far, among the main challenges that marine fishery scientists are currently facing^5^. Therefore, the efficacy assessment and enforcement of conservation/management measures that, at the same time, ensure short-time fishery benefits while preserving ecosystems’ ability to produce goods and services, are crucial issues to be addressed jointly by scientists, stakeholders and policy makers. In this regard, FPAs are recognized as fundamental parts of the multifaceted toolkit available for the conservation of heavily exploited species^12,14,17,22,23,30^. The effects of overfishing on single species may be reversible, but the actual time required for recovery is generally long^31^. Lobsters, because of their moderate mobility are reported to be a good model for studying response to fishing restrictions in FPAs^28^.

Previous experiences aimed at enhancing stocks of the European spiny lobster *P*. *elephas* based on grow-out and restocking through large quantities of *puerulii* (*i*.*e*., post larval stage) did not succeed^12^. To our best knowledge, our study is the first one ever addressing the efficacy of European spiny lobster restocking in spatially replicated FPAs, using below legal-size specimens obtained by local fisheries outside FPAs and therein reallocated through a CFR program.

Available scientific literature does provide several examples of natural recovery of decapod crustaceans within temperate MPAs, actually not aimed at fishery enhancement as a main goal. Results from these studies showed a great variability depending on target species and geographical locations. Within the Mediterranean *scenario*, tag-recapture experiments on *P*. *elephas* performed in the Columberete Island Marine Reserve in Spain documented a net benefit of 10% in biomass increase after 17 years of protection from neighboring fishing grounds^32^. Another study monitored trends in density and biomass for 25 years; in this study, which actually documents natural recovery occurred from the 10^th^ to the 25^th^ year, showed how CPUE in biomass more than doubled, with considerable fluctuations among years^28^.

From other parts of the world, several responses of populations of *Jasus edwardsii* (Hutton, 1875) are documented in New Zealand waters, displaying highly variable responses to protection, with a few showing a rapid recovery within MPAs borders and others showing little response even after a decade of protection^33^. *Palinurus interruptus* (Randall, 1840), in California, developed significant and considerable benefits after 5–6 years of reserve establishment, with fishing yield increased by 4 to 6 times within MPA. Two years of protection within MPAs aimed at recovering stocks of South African rock lobster *Jasus lalandii* (Milne-Edwards, 1837) did not succeed, with lobster abundance being not always higher than neighboring fishing grounds.

We show that, in all of the three FPAs, lobsters’ stocks after 5 years of protection and active restocking were significantly higher than those at the beginning of the experiment (*i*.*e*., between years 0–1 and 4–5). Our result, in addition, suggests that in our case timing of recovery was much faster than in other experiences based on a mere conservation without active restocking. Having included in the experiment the FPA of Castelsardo which was not interested by restocking, although not spatially replicated, does allow us to emphasize the positive effect provided singularly by protection and by the combination of protection *and* restocking, with the latter being crucial for the observed swift recovery (Fig. 4).

*P*. *elephas* shows generally short-distance movements and poor homing skills when dislocated far (*i*.*e*., > 0.5 Km) from the capture point^34,35^ but, at the same time, its migration is strongly density-dependent^28^. Thus, when below legal-size lobsters released within the FPA grow and the carrying capacity of the area is reached (promoting density dependent emigration), a ‘*fishery spillover’ (sensu* Di Lorenzo *et al*.^26^), is expected. In two out of the three FPAs (Bosa and Castelsardo) the drop in CPUE inside the FPAs at year 5 was accompanied by an increase or persistence of high CPUE values outside (Fig. 2).

All FPAs and neighbouring fishing grounds witnessed an increase in density and biomass of specimens belonging to small size classes, as confirmed by the demographic analyses, and the decreasing mean size of individuals (Supp. Figs 1, 2 and 3). However, Bosa FPA, showed significant positive effects also in terms of biomass (CPUE) of individuals that could be landed (>90 mm in CL). The fact that only Bosa showed significant increase in biomass of legal lobster could be ascribable to the higher depletion status at the beginning of the experiment, compared to other FPAs. Indeed, Bosa FPA showed the lowest value of both CPUE and nCPUE at the beginning of the experiment and, at the same time, the most positive effect on lobsters’ biomass (LnR = 1.71 ± 0.45; 95% CI).

At the end of restocking, the spillover effect in Buggerru was less intense (ca. 30 and 40% increase in biomass and density, respectively), despite being the FPA with the highest increase from the initial status. In this regard, it worth noticing that the extension of the Buggerru FPA is 40–100% larger than that of the two other FPAs. This could have implied that, at the end of the fifth year, the carrying capacity in Buggerru could have not been reached yet, and we could have expected even larger density and biomass values both inside and outside FPA. This, however, was not the case, indicating that other, undefined, factors intervened masking the eventual spillover effects in this FPA.

In this context, we must notice also that our contentions refer to ‘*fishery spillover*’ (the spillover mediated by density dependent migration outside the protection area), that represents one the two components of spillover promoted by marine reserves, the first to be operatively detected, and the most tangible for stakeholders^25,26^. The ‘*ecological spillover’* (the spillover mediated by the enhanced production of biomass inside the protection area) occurs over longer time scales and was not considered in our study. However, it worth pointing out the benefits that FPAs provided on the reproductive potential of the whole population. The relative abundance of large individuals (>105 mm in CL; Supplementary material), which contribute the most to the population reproductive potential^36^, showed an increase within different FPAs. This increase has to be added to the portion of spiny lobsters already within FPAs and those reallocated therein, despite cumulatively contributing less to the whole potential of the population^36^. Outside FPAs, on the contrary, large individuals are systematically caught as they have reached the minimum legal size to be landed, impairing the reproductive potential of the whole population inhabiting neighbouring fishing grounds. In this case, FPAs benefits are mostly reflected by a swift increase in both density and biomass of smaller individuals (Supplementary material).

From a management perspective, policymaking should consider the time-scale for tangible benefits for fishermen, which is the social category facing the most relevant sacrifices in terms of restrictions. The initiative here described, carried out tightly by researchers and fishermen under a CFR program, allowed obtaining a positive and tangible increase of catches in each of the three FPAs and nearby waters within 5 years, with sometimes significant increase also in biomass of lobsters that can be landed (>90 mm in CL; *i*.*e*., outside Bosa FPA). Fishery spillover, here estimated in terms of increase in catches (both biomass and density) in fishing grounds comprised in a maximum of 5 nm range from the centre of each FPA, was observed since the beginning of the CFR experience, allowing stakeholders to witness their own crucial role as ‘lobster-farmers’ rather than fishermen into the recovery of depleted stocks.

The pilot FPA of Su Pallosu, despite not being spatially replicated, provides useful insights regarding the long-term efficacy of restocking. Our results show how, after restocking ceased and protection was kept in force, the population approached a self-sustained persistence within FPA borders. The Su Pallosu FPA shows how, once restocking has been stopped, decrease in CPUE increased after a short period, sustained by the population itself and not by the introduction of new wild juveniles. Available literature could explain the observed pattern through the fact that large individuals, those mostly contributing to the reproductive potential of the population, are likely to be sedentary within FPA borders^37^. Indeed, such large individuals do have increased chance to reproduce within FPAs and juveniles are likely to be responsible of the increased biomass within FPA, after >5 years when restocking was stopped. In addition, a decrease in CPUE is observed in the last year of restocking also in the more recently established FPAs.

Final result of the restocking experiment here described consisted in a significant increase in lobsters’ biomass and density (here estimated through CPUE and nCPUE) inside and outside FPAs, either at the local (*i*.*e*. in each FPA) and cumulatively, in all FPAs, within 5 years. Under this perspective, we complain that similar CFR programs can ideally be transferable to other regions and that reserve/no take-areas networks associated with active restocking practices could promote the sustainability of *P*. *elephas* fishery.

However, it worth pointing out how Sardinia, beside a restricted fishing period (see methods) does not have a limit in the number of fishing boats operating outside FPAs, nor *quotas* in terms of weight or number of individuals per each fishing boat, nor a limit in fishing effort (in terms of length of deployable trammel nets). Notwithstanding the restrictive measures adopted since 90 s, catches kept decreasing across decades until today, arising the demand and the market price of *P*. *elephas*, fostering the black market of under-sized individuals as side-effect.

In this perspective, despite the tangible benefits provided by this experience, managers should consider additive measures to FPAs and restocking, such as limited number of pieces of net or boats, that could further enhance the recovery of depleted stocks and their more sustainable exploitation on the long term.

## Material and Methods

### Palinurus elephas fishery in the Mediterranean Sea

The European spiny lobster *Palinurus elephas* is a long-lived and slow-growing species typical of temperate waters, widely distributed in the Mediterranean Sea and NE Atlantic Ocean^38^.

The *P*. *elephas* stocks’ decline, as for most other stocks worldwide, has been determined by the development of more powerful fishing fleets and more efficient fishing gears: the former supported by increasingly efficient technologies, the latter gradually shifting from traps to trammel nets^38^.

According to FAO landings data (Global Capture Production Summary; the only available official data) compared to the first half of the 20^th^ century, a drastic decrease in landed tons occurred from 1980s for all Mediterranean and Atlantic countries involved in *P*. *elephas* fishery (*i*.*e*., from 8710 to 4242 tons per year)^39^.

Sardinia, Corsica, Balearic Islands and Sicily do, overall, host the most numerous fleets (>200 vessels and >1500 fishermen involved) and do represent the majority of landings in the Mediterranean (*i*.*e*., ca. 70% of landings reported in 2000, according to FAO data). Up to date, Sardinian regulation (which was in force since 1990s) imposes a restricted fishing period (from March 1^st^ to August 30^th^, according to a national law), and a minimum legal size of 90 mm in CL (or 26 cm in total length), according to the European regulation 1967/2006. The size of 77 mm in CL represent the average size for females of *P*. *elephas* to be functionally mature^36^, thus, the minimum size of 90 mm represents a size were female specimens are supposed to be 1 or 2 years older than the first maturity size^36,38^.

### Establishment of no take reserves and study design

The study was conducted in the framework of two restocking program of the European spiny lobster in Sardinian sea, promoted and funded by the Autonomous Region of Sardinia (RAS) (Regional Law No. 776 of 6-5-1998 and N82069/DecA/84, 8.11.2009)^29^.

FPAs were officially established by RAS and thus protected by regular surveillance of Coast Guard and other police forces operating at sea, to prevent any fishing activity within FPAs. Local fishermen provided an additional supervision to the already in force FPAs’ surveillance. All areas were designed in accordance with local fishermen communities with the aim to protect *P*. *elephas* from overexploitation, contrast the black market of below legal-size specimens and, possibly, enhance nearby catches through spillover effects.

Three FPAs were established in 2010 in the northern (Castelsardo), northwestern (Bosa) and southwestern (Buggerru) coasts (Fig. 1), over rocky bottoms characterized by coralligenous and pre-coralligenous habitats^40,41^. Extension and depth range of the three FPAs are reported in Table 1. The extension was established in order to have FPAs boundaries placed at minimum 1.3 nautical miles from the release point of specimens used for restocking. Such a distance represents the mean annual distance covered by wild adult specimens of *P*. *elephas*^29,35,42^. Restocking was effectively carried out during five consecutive years, starting from 2011 up to 2015. Specimens used for restocking were caught during commercial fishing in areas nearby and outside the FPAs. All specimens used for restocking were measured by the researchers and, if below the legal size, tagged immediately upon capture using individually numbered T-bar anchor tags (inserted dorso-laterally using an appropriate pistol), and released at the center of the reserve at the end of the fishing day. RAS also provided through the abovementioned laws all required permission for carrying sub-legal sized specimens’ onboard vessels involved in the CFR program and perform tagging during the experiment.

To test the hypothesis by which the establishment of no take reserves replenished with below legal-size specimens can induce a significant increase in density and biomass within FPAs, inducing spillover within a relatively short-term period (*i*.*e*. within five years), we measured annual Catch per Unit Effort of European spiny lobsters obtained with trammel-nets inside and outside each of the three reserves before their establishment in 2010, and, annually, for the subsequent 5 fishing seasons (March 1^st^–September 30^th^). Catch Per Unit of Effort in terms of density (nCPUE) and biomass (CPUE) was calculated as number of individuals and grams of *P*. *elephas* per piece of net (*i*.*e*., 50 linear meters of net).

In addition, to test if observed effects do persist also when restocking in FPAs is stopped, we kept measuring CPUE in the pilot FPA in Su Pallosu for 7 years after restocking ceased.

The pilot study^29,37,43^ reports data from 1998 to 2005, collected with the same protocol proposed in the newly established FPAs, comprising the 5 years of restocking and 3 years after it has ceased. We here report the continuous of the experiment in the pilot area, up to 2013, covering a total of 15 years.

Also, to discriminate the effect provided by active restocking from the effect of protection provided by the FPA, we confronted trends in CPUE and nCPUE of the Castelsardo FPA with another control area established in Castelsardo, with the same extension, environmental features and enforcement of the Castelsardo FPA, but restocking was not performed.

Surveys inside FPAs were performed using trammel-nets, which is documented as efficient and successful tool for experimental surveys aimed at assessing spiny lobsters stocks^28^. Used trammel nets had nominal mesh size ranging from 50 to 73 mm; the same type of gear was used both for surveys inside and outside FPAs. CPUE data from outside the FPAs were provided by the local fishermen, who, assisted by the researchers, carried out the experimental surveys also inside the FPAs. All specimens caught inside FPAs, comprising adults were released back into the sea after biometric parameters were retrieved.

Data from outside FPAs were comprised within 5 nautical miles from the FPA center. Such distance has been set as it exceeds the theoretical maximum distance that a spiny lobster could reach from the release point at the center of the FPA after 5 years^29,35,42^. Number of replicate surveys conducted inside and outside each FPA during the five years is reported in Table 1.

### Data analysis

We tested our hypothesis through non-parametric permutational analyses of variance ‘PERMANOVA’ (software PRIMER 6+, Plymouth Marine Laboratory), based on ‘Euclidean distance’ similarity matrixes of non-transformed data. The experimental design included 2 orthogonal fixed factors: time (with six levels: year 0, 1, 2, 3, 4, 5) and FPA (with 2 levels: inside *vs*. outside), with a variable number of replicates (Table 1).

To avoid any bias due to the different ecological conditions characterizing the three FPAs, the analyses were performed separately for each FPA. Post-hoc tests were also conducted to assess: (i) differences in CPUE and nCPUE between inside and outside the FPA across the five years and (ii) differences in CPUE and nCPUE across sampling years, within inside and outside the FPA, separately.

In addition, to test whether active restocking could provide significant fishery benefits outside FPAs, a routine including the single factor ‘time’ (with six levels: year 0, 1, 2, 3, 4, 5) using density and biomass of individuals that could be landed, was performed.

For each sampling year, forest plots obtained using Log-transformed ratios of annual mean CPUE and nCPUE inside (impact) and outside (control) FPAs were used to visualize patterns of change across years per each FPA, either separately or cumulatively.

Forest plots were performed using R Studio (R Development Core Team, 2016), through the meta-analysis packages “metafor”^44^ and “robumeta”^45^; the script has been created by Quintana (2015)^46^ and modified using the Log-transformed ratios of means as effect size.

## Supplementary information

Supplementary material
