# Supplementary material for: European spiny lobster recovery from overfishing enhanced through active restocking in Fully Protected Areas
Source: Sci Rep. 2019 Sep 10;9:13025. doi: 10.1038/s41598-019-49553-8 (PMC6737030; doi:10.1038/s41598-019-49553-8)
Supplement: Supplementary file 1 — Supplementary material [file 41598_2019_49553_MOESM1_ESM.docx]

**European spiny lobster recovery from overfishing enhanced through active restocking in Fully Protected Areas**

Alessandro Cau^1,3🖂^, Andrea Bellodi^1,3^, Rita Cannas^1,3^, Maurizio Fois^1^, Paolo Guidetti^2,3^, Davide Moccia^1,3^, Cristina Porcu^1,3^, Antonio Pusceddu^1,3^& Maria C. Follesa^1^

^1^Dipartimento di Scienze della Vita e dell’Ambiente - Università di Cagliari - Via Tommaso Fiorelli 1, 09126 Cagliari, Italy.

^2^Université Côte d’Azur, CNRS, FRE 3729 ECOMERS, Parc Valrose 28, Avenue Valrose, 06108 Nice, France.

^3^Consorzio Interuniversitario per le Scienze del Mare, CoNISMa, Piazzale Flaminio 9, 00196 Rome, Italy.

**Supplementary Figure 1.** Size distribution of P. elephas in the Bosa FPA. Graphs in blue represent the size distribution outside FPA across years and orange graphs represent size distribution inside FPA.

**Supplementary Figure 2.** Size distribution of P. elephas in the Buggerru FPA. Graphs in blue represent the size distribution outside FPA across years and orange graphs represent size distribution inside FPA.

**Supplementary Figure** **3.** Size distribution of P. elephas in the Castelsardo FPA. Graphs in blue represent the size distribution outside FPA across years and orange graphs represent size distribution inside FPA.
